# Post-COVID-19 cardiological alterations

**DOI:** 10.1590/1516-3180.2021.139628062021

**Published:** 2021-08-30

**Authors:** Alexandre de Matos Soeiro, Paulo Manuel Pêgo-Fernandes

**Affiliations:** I MD, PhD. Attending Physician, Division of Cardiology, Instituto do Coracao, Hospital das Clinicas HCFMUSP, Faculdade de Medicina, Universidade de Sao Paulo, Sao Paulo, SP, BR.; II MD, PhD. Full Professor, Thoracic Surgery Program, Instituto do Coracao, Hospital das Clinicas HCFMUSP, Faculdade de Medicina, Universidade de Sao Paulo, Sao Paulo, SP, BR; Director, Scientific Department, Associação Paulista de Medicina, São Paulo (SP), Brazil.

Since the end of the year 2019, the world has experienced a rapid and progressive public health emergency. The disease caused by the virus SARS-CoV-2 (severe acute respiratory syndrome coronavirus 2), named COVID-19 (coronavirus-19), has been shown to be a broad-spectrum and unpredictable condition in which while some patients remain practically asymptomatic, others may present a disease course involving severely compromised lungs. This is the greatest cause of the morbidity and mortality attributed to this disease.^1^^,^^2^

However, early in the course of COVID-19, the disease has been shown to have a wide-ranging and potentially alarming interface with the cardiovascular system. Angiotensin-converting enzyme 2 (ACE2) receptors have been shown to have a direct connection with viral pathogenesis, and these may form the cellular gateway in type 2 pneumocytes, macrophages and cardiomyocytes.^1^ Thus, patients with cardiovascular diseases have been found to be more susceptible to severe forms of COVID-19. Hypertension, arrythmias, myocardiopathies and coronary artery disease are among the main comorbidities present in patients who are critically ill with COVID-19. Likewise, myocardial injury has been shown to be a potential marker for mortality in COVID-19. The mechanisms for cardiovascular lesions that have been proposed remain incompletely established but it has been suggested that these may involve direct damage to cardiomyocytes, systemic inflammation, interstitial fibrosis of the myocardium, immune-mediated response to interferon, excessive cytokine response by T cells, endothelial dysfunction, destabilization of coronary platelets and hypoxia.^1^^,^^2^

Patients with cardiovascular diseases, and particularly hypertensive patients, present morbidity rates of up to 10.5% after becoming infected with COVID-19. A recent analysis on 5,700 patients hospitalized in New York showed that the most prevalent comorbidities were hypertension (57%), obesity (42%) and diabetes (34%).^2^ Another study conducted in China among 1,527 patients hospitalized with COVID-19 showed that the prevalences of hypertension, cardiovascular disease and diabetes were, respectively, 17.1%, 16.4% and 9.7%. These prevalences were two to four times higher in patients with severe COVID-19 than in mild cases of the disease.^3^

As mentioned earlier, another finding relating to COVID-19 mortality has been the presence of myocardial injury. Elevation of troponin levels has been shown to be significantly related to higher mortality and to cardiac arrhythmias. Increased levels of this marker occur more frequently in individuals with chronic cardiovascular diseases than in previously healthy individuals. Higher prothrombotic and inflammatory activity and hypoxia contribute to occurrences of myocardial injury. However, presence of myocarditis, stress-induced cardiomyopathy, acute heart failure and direct lesions of cardiomyocytes also contribute to these occurrences. Even conditions that are not directly related to the heart but common in COVID-19 can lead to increased troponin levels. These may include pulmonary embolism, sepsis and critical states among patients. Shi et al.^4^ showed that the rate of occurrence of myocardial injury among patients hospitalized with COVID-19 was 19.7%, and that this had a direct positive correlation with disease presentations of greater severity. Another similar study showed that myocardial injury occurred in up to 41% of the patients with moderate-to-severe conditions. Furthermore, the mean troponin level among patients who died was 40.8 ng/dl, versus 3.3 ng/dl among those who survived.^4^

Among the cardiovascular manifestations relating to COVID-19, cardiac arrhythmias were observed in 16.7% of the hospitalized patients: 7% of those who did not require intensive care observation and 44% of those who were admitted to an intensive care unit. The manifestations ranged from benign arrythmias such as atrial fibrillation to atrioventricular blockage and ventricular tachycardia/fibrillation. Metabolic dysfunctions, inflammation and activation of the sympathetic nervous system are thought to be the main predisposing factors for heart rhythm alterations.

Heart failure has been reported in up to 23% to 33% of patients hospitalized with COVID-19 in China. It was observed in 52% of the patients who died, versus in 12% of those who survived. Myocardial injury can be caused both by myocarditis and by imbalance of demand/consumption.^2^^,^^5^ Specifically, acute myocarditis has been presented in some samples as the cause of death in up to 7% of patients with COVID-19, and it can be presented in a fulminating manner. However, such diagnoses are not necessarily confirmed, and this percentage may have been overestimated. Two particular features are now being evaluated within this context. Firstly, some cases of myocarditis among patients with COVID-19 may be caused by infections due to other concomitant viruses. Secondly, while acute myocarditis may be present at the most critical moment within the course of COVID-19, some patients have an autoimmune reaction and present subacute myocarditis several weeks after the initial infectious event. All of these suppositions are being studied.^6^

Myocardial ischemia has become another concern in relation to presentations of COVID-19. The various mechanisms for endothelial dysfunction, the prothrombotic state, the inflammation and the destabilization of atherosclerotic plaques consequently lead to potential for these patients to remain more exposed to acute coronary events. The real incidence of these phenomena continues to be uncertain. However, it is known that most infarcts are of type 2, due to imbalance between demand and consumption. Greater difficulty in found in treating patients with type 1 infarct, for whom the time to implement catheterization, the antithrombotic therapy and the safety of the team involved need to be assessed in combination, in order to achieve the best management for the patient.^2^

Lastly, in addition to the acute cardiovascular diseases that are present during the course of COVID-19, we are now starting to deal with the sequelae: these are sometimes irreversible or have a slow and difficult recovery process. Many survivors of severe COVID-19 continue to complain of symptoms for long periods, even after their discharge from hospital. It remains unknown whether this might represent a new post-COVID syndrome. Approximately 21.4% to 43.4% of the patients continue to report having dyspnea two to six months after becoming infected with COVID-19. Palpitations and chest pain are reported by, respectively, 9% to 32% and 5% to 44% of the patients. Around 58% of the patients who present cardiovascular manifestations during the acute phase of COVID-19 continue to show cardiological sequelae on cardiac magnetic resonance imaging 50 days later, with reduction of the ejection fraction and presence of edema and myocardial fibrosis.^7^

In this light, instituting cardiovascular rehabilitation programs now takes on fundamental importance. In addition to the patients with COVID-19 who have presented severe cardiological sequelae, it needs to be borne in mind that all other patients with cardiopathies but without COVID-19 infection have also reduced their physical activity levels consequent to social isolation. It has been estimated that individuals have reduced their physical activity by up to 25% during the pandemic, in relation to their previous levels. Some centers in the United States have implemented in-person and telemedicine rehabilitation systems for these patients, with priority given to those with conditions of greater severity, and have obtained good follow-up results.^8^

Thus, we conclude that there is now better understanding of COVID-19 and its cardiovascular manifestations. We now know the extent to which the presence of cardiovascular comorbidities and the cardiological manifestations of COVID can worsen the prognosis. Nonetheless, many questions regarding their physiopathology and treatment remain open, to be targeted in future clinical studies.
